# Volunteers’ compassion fatigue, compassion satisfaction, and post-traumatic growth during the SARS-CoV-2 lockdown in Spain: Self-compassion and self-determination as predictors

**DOI:** 10.1371/journal.pone.0256854

**Published:** 2021-09-01

**Authors:** Rosaura Gonzalez-Mendez, Matilde Díaz

**Affiliations:** Departamento de Psicología Cognitiva, Social y Organizacional, Universidad de La Laguna, La Laguna, Canary Islands, Spain; University of Milan, ITALY

## Abstract

Volunteers have played an important role by supporting essential services that have been overwhelmed during the most critical moments of the SARS-CoV-2 pandemic. Hence, nonprofit organizations may be interested in preventing negative consequences of these volunteers’ exposure to potentially traumatic events. The aim of this cross-sectional study was twofold. First, to examine to what extent self-compassion and self-determination would contribute to differentiating between volunteers with different levels of compassion fatigue, compassion satisfaction, and post-traumatic growth. Second, to identify the best predictors of the most extreme levels of each outcome. Participants were 211 Spanish Red Cross volunteers (60.7% women), who completed a survey. They were separately classified into three groups (low, medium, and high) according to the 33rd and 66th percentile scores on each outcome (compassion fatigue, compassion satisfaction, and post-traumatic growth). Univariate analyses of variance and post-hoc comparisons revealed that self-compassion and self-determination contributed differently to distinguishing between levels of each outcome. Volunteers lowest in compassion fatigue stood out for showing fewer non-compassionate strategies and more mindfulness than the other groups. Moreover, those higher in satisfaction compassion also showed lower use of unhealthy strategies and higher scores in all other predictive variables. Volunteers highest in post-traumatic growth showed higher self-kindness and satisfaction of all psychological needs. Binary logistic regressions allowed for the identification of predictors of belonging to the most extreme groups. The protective factors may be useful to guide volunteers’ self-care and help them thrive in the face of critical service demands.

## Introduction

Spain has been one of the European countries most affected by the SARS-CoV-2 pandemic [1, 2], which has strained the resistance of both the healthcare system and other essential services. In this context of crisis, nonprofit organizations have covered many urgent social demands that arose with the start of the pandemic (e.g., support for emergency services, care for the elderly, basic supplies for those in need). They also work with the groups who are most vulnerable to the outbreak, who are often off the radar in most surveys. Volunteers have played a key role in the aid effort, dealing directly with people’s suffering. This means that nonprofit organizations may be interested in preventing negative psychological consequences of volunteers’ exposure to highly stressful and potentially traumatic events. However, there is still little guidance for volunteers’ self-care and resilience [3], especially in a pandemic context.

Helping suffering people may lead aid professionals to experience compassion satisfaction [4] and even thrive [5–7]. For instance, effective patient care and thriving have been found in health professionals with high self-determination [6]. However, most research has focused on unwanted changes to emotions, cognitions, and behaviors [8]. Compassion fatigue [9] is one aftermath with the potential to negatively affect the well-being and labor of professionals who deal with distressing populations [10, 11]. While burnout can have an effect on any professional who unsuccessfully manages chronic work stress [12], compassion fatigue emerges specifically in those who deal with the suffering of others [13]. In addition to reducing compassion satisfaction, this debilitating condition is accompanied by symptoms such as exhaustion, hopelessness, avoidance, and numbing. It has been extensively studied in health and human service providers [13, 14], but rarely examined in volunteers [15]. Available studies on this latter group indicate that prevention requires building a supportive organizational environment [5, 15] and reinforcing specific protective factors that contribute to resilience [16, 17]. The emergence of the concept of resilience has meant that psychologists have changed their focus from looking for the origin of psychological pathologies to identifying the assets and resources that promote healthy functioning [18]. Hence, this framework is pertinent when the spotlight is on prevention.

Most researchers define psychological resilience as a positive adaptation in the face of adverse events [19]. However, it can also be understood in a broader sense, when it involves thriving through developing new skills, strengthening relationships, and broadening perspectives [20]. This latter meaning of resilience connects with the construct of post-traumatic growth [21], one of the outcomes analyzed in this study along with compassion fatigue and compassion satisfaction. Post-traumatic growth has been defined either as an enduring positive psychological change experienced after trauma or challenging life circumstances [22, 23] or as a coping strategy with an adaptive psychological function [24]. However, both models implicitly assume that post-traumatic growth is a positive and adaptive phenomenon [21]. Post-traumatic growth is often used as an indicator of resilience, because these two constructs are empirically related, even though they are not identical [25, 26]. Being resilient does not require one to experience traumatic events, nor does it necessarily involve thriving. Hence, resilience may be considered a broader construct than post-traumatic growth.

The explanation of compassion fatigue is still under discussion. On the one hand, researchers have assumed that compassion fatigue occurs when the compassionate effort demanded surpasses the restorative capabilities, thus provoking feelings of impotence [27]. On the other hand, neuroscience has highlighted that this type of fatigue is not due to excessive compassion, but to a blurred self-other distinction [28, 29]. In this regard, compassion fatigue would be seen as a strong aversive response accompanied by the desire to withdraw from a situation to protect oneself from negative feelings [29]. The proposals for preventing this unwanted form of aftermath differ for each approach, and involve either increasing compassion satisfaction to balance the ratio between both dimensions [5] or undergoing training in true compassion to prevent ‘empathic distress fatigue’ [28]. However, both interpretations seem to share the idea that cultivation of self-compassion is among the critical skills to be empathic, compassionate with others, and resilient [28, 29].

Bearing all this in mind, we wanted to analyze whether the three outcomes mentioned above (compassion fatigue, compassion satisfaction, and post-traumatic growth) benefited from two protective factors, self-compassion and self-determination.

Research has identified self-care among the protective factors that contribute to reducing compassion fatigue among professionals [7]. However, it refers to an imprecise set of “successful thoughts and actions that result in improving or maintaining one’s good physical and mental health and a general sense of personal comfort” (p.11) [3]. In this regard, the self-compassion construct [30] allows for the operationalization, in a more precise way, of some specific strategies that have proven healthy or harmful. Although these strategies are originally connected with different theoretical models [31], Neff incorporated them into the same construct and provided evidence that both compassionate and reduced uncompassionate self-responding contribute to healthy psychological functioning [32, 33].

Self-compassion represents, in fact, a positive way of relating to oneself in moments of failure, perceived inadequacy, or life difficulties [30, 32, 33]. It entails being understanding with oneself (Self-kindness), recognizing that life difficulties are part of the shared human experience (Common Humanity), and keeping an emotionally balanced approach in the face of negative experiences (Mindfulness). Moreover, self-compassion protects against the negative consequences of harmful strategies such as disapproval (Self-judgment), feeling of disconnection from others (Isolation), and amplification of personal suffering (Over-identification).

Being self-compassionate has been found to reduce the impact of trauma exposure [34] and to increase positive health outcomes and well-being [30, 35]. It has also been linked to reduced functioning of the sympathetic nervous system and lower inflammatory activity after stress [32]. Importantly, self-compassion is recognized as a personal strength that may be acquired through practice, contributing to the well-being of helping professionals themselves [11, 36]. By contrast, non-self-compassionate strategies have been associated with the denial of self-related needs and a pathological concern for others, which leads to emotional and interpersonal difficulties and a lack of well-being [37].

As indicated above, high self-determination has been related to thriving in health professionals [6]. Following this line of analysis, we also tested whether self-determination could be consistently related to self-compassion, as well as discriminating between volunteers with different levels of compassion fatigue, compassion satisfaction, and post-traumatic growth. As conceptualized by Deci [38], ‘true self-esteem’ is an integrated and healthy sense of self that emerges from the satisfaction of three basic psychological needs for autonomy, competence, and relatedness. Autonomous self-esteem, resulting from the ongoing satisfaction of these three basic needs, has been positively related to more effective performance, psychological health, and thriving [39, 40]. Conversely, failure to meet these needs leads one to develop contingent self-esteem and poorer motivation, which makes people dependent on particular outcomes or social approval (e.g., reward or punishment, approval motive). To be precise, partial satisfaction of competence and relatedness needs, but not of autonomy, has been related to rigid functioning and diminished well-being. In addition, being unable to satisfy all three needs has been associated with poor functioning and unhealthy symptoms [39]. From this point of view, organizational contexts may differ in their contribution to enhancing a volunteer’s autonomous self (i.e., self-determination) through the satisfaction of these three basic psychological needs. Hence, knowing to what extent they enhance self-determination could help organizations prevent compassion fatigue among volunteers, as well as boosting compassion satisfaction and thriving in the face of the pandemic.

### The current study

The lockdown in Spain lasted from March 14th to June 21st, whereas data collection started on June 24th and ended on July 14th. According to data provided by the Red Cross, the number of volunteers who joined in 2020 almost doubled the number of active volunteers. The organization currently has around 175,000 volunteers throughout Spain. The RESPONDE program [41] managed to mobilize approximately 3,300 volunteers in the Canary Islands (1,693 on the island of Tenerife, where data were collected), of whom 43% were new volunteers.

During the SARS-CoV-2 lockdown, Red Cross volunteers played a key role by supporting essential services, which involved a greater exposure to potentially traumatic events and likely an increased risk of compassion fatigue. Although enhancing resilience has been suggested as an effective way to prevent compassion fatigue among professionals [3], there are few studies focused on volunteers’ resilience that offer guidance on how to prevent this difficulty. According to this, the first objective of this study was to analyze to what extent higher self-compassion (as shown by a higher frequency of self-compassionate strategies and a lower frequency of non-self-compassionate ones) and self-determination (sustained by the satisfaction, through volunteering, of basic psychological needs) would help differentiate those volunteers with low compassion fatigue and high compassion satisfaction and post-traumatic growth. Moreover, a second objective was to identify the best predictors of the most extreme (i.e., low and high) groups according to each outcome. With some exceptions [37], these protective factors have not been analyzed together, and much less with regard to the three outcomes examined in this study.

As indicated above, higher self-compassion reflects different positive ways of relating to oneself that are healthy (self-kindness, common humanity, and mindfulness), which have been shown to be protective in stressful situations and contribute to increasing positive health outcomes and the well-being of health and social service providers. Hence, a first question raised was whether volunteers with a lower level of compassion fatigue and higher compassion satisfaction and post-traumatic growth would report more frequent use of self-compassion strategies during the crisis provoked by the lockdown. To be precise, we expected that those who indicated a lower level of compassionate fatigue, as well as higher levels of compassion satisfaction and post-traumatic growth, would show greater use of compassionate strategies (self-kindness, common humanity, and mindfulness) than the other groups (Hypothesis 1).

Conversely, lower self-compassion is indicated by unhealthy strategies (self-judgement, isolation, and over-identification), which contributes to worsening the response to stressful situations and a decrease in well-being. Therefore, the second question to answer was whether those volunteers with worse outcomes would show a more frequent use of non-self-compassionate strategies. In this case, we expected that those who scored higher in compassion fatigue, as well as lower in compassion satisfaction and post-traumatic growth, would show a higher use of non-self-compassionate strategies (self-judgement, isolation, and over-identification) than the other groups (Hypothesis 2).

Moreover, the satisfaction of basic psychological needs (autonomy, competence, and relatedness) has proven to be positively related to more effective performance, psychological health, and growth [39, 40]. According to the self-determination approach, the satisfaction of these basic psychological needs would guarantee maintaining an autonomous self, less dependent on adverse circumstances. In this case, the question raised was whether this approach would be useful to discriminate between the volunteers’ response during the lockdown. We expected that those who indicated a lower level of compassionate fatigue, as well as higher levels of compassion satisfaction and post-traumatic growth, would show a greater satisfaction of the basic psychological needs (autonomy, competence, and relatedness (Hypothesis 3).

## Materials and methods

### Participants

Participants were 211 Spanish Red Cross volunteers (60.7% women) with ages ranging from 18 to 69 (*M* = 42.8, *SD* = 12.0). The time spent on volunteering ranged from 1 month to 43 years (*M* = 4.1, *SD* = 7.7). Most attended urgent demands during lockdown, which meant assisting vulnerable groups (lonely elderly, homeless, or people with economic difficulties) (51.6%), health emergencies (17.1%), food management and distribution (12.8%), and telephone attention (9.9%), among others.

### Design and procedure

A cross-sectional design was used to examine the data collected using an online survey. We analyzed three outcomes (compassion fatigue, compassion satisfaction, and post-traumatic growth) and different predictive variables grouped into two potential protective factors (self-compassion and self-determination).

The study received the approval of the local leaders of the Spanish Red Cross (Tenerife, Spain) and the Institutional Review Board of the corresponding author’s university (CEIBA2020-0422). The objective of the study was first explained to volunteers by the staff of different local services of the Red Cross Organization. They also sent the volunteers the link to the survey. Those who agreed to participate were asked to sign their informed consent after being informed of the objectives of the study and before responding to the survey. The anonymity of their responses and the confidentiality of the data were guaranteed at all times. We also stressed the independence of the research team with respect to the organization. Data collection was carried out shortly after lockdown in Spain ended, over two weeks.

### Measures

#### Compassion fatigue and compassion satisfaction

Each of these constructs was evaluated using the subscales of the Spanish version of the Professional Quality of Life Scale, R-IV (ProQOL) [42]. While the 10-item subscale of Compassion Fatigue measures the negative consequences of being affected by adverse experiences of people with whom one works (e.g., ‘I think that I might have been affected by the traumatic stress of those I help’), the 10-item subscale of Compassion Satisfaction assesses the pleasure derived from being able to do your work helping others well (e.g., ‘my work makes me feel satisfied’). The response options in both cases were (0 = *never*, 1 = *rarely*, 2 = *a few times*, 3 = *somewhat often*, 4 = *often*, and 5 = *very often*). The internal consistence was tested using Cronbach’s alpha, reaching values of .64 for compassion fatigue and .83 for compassion satisfaction.

#### Post-traumatic growth

We used a Post-traumatic Growth scale from the Resilience Portfolio Measurement Packet [43]. This nine-item scale is used to measure thriving after adversity, since it evaluates increased strengths, appreciation of life and people, spiritual change, etc. The items were adapted to assess the positive changes that the participants attributed to their experiences in volunteering during the lockdown (e.g., ‘I have changed my priorities about what is important in life’, ‘now I know that I can handle hard times’). The response options ranged from 4 (*mostly true*) to 1 (*not true*). Cronbach alpha was .91.

#### Self-compassion

Self-compassion was assessed using the Self-Compassion Scale–Short Form [44]. This short version consists of 12 items grouped into six strategies (two items for each), which have shown adequate reliability in previous research [45]. Three reflect healthy strategies: *Self-kindness* (e.g., ‘when I’m going through a very hard time, I give myself the care and tenderness I need’); *Common Humanity* (e.g., ‘I try to see my failings as part of the human condition’); and *Mindfulness* (e.g., ‘when something upsets me, I try to keep my emotions in balance’). By contrast, the other three strategies are clearly unhealthy: *Self-judgment* (e.g., I’m disapproving and judgmental of my flaws and inadequacies’); *Isolation* (e.g., ‘when I fail at something that’s important to me, I tend to feel alone in my failure’); and *Over-identification* (e.g., ‘when I’m feeling down I tend to obsess and fixate on everything that’s wrong’). Response options of all the sub-scales ranged from 1 (*almost never*) to 5 (*almost always*). Internal consistency of each sub-scale was calculated using Cronbach’s alpha, reaching the following values: self-kindness (α = .83), mindfulness (α = .78), isolation (α = .71), and over-identification (α = .83). The measures of common humanity (α = .51) and self-judgment (α = .50) were discarded for analyses due their low internal consistency.

#### Satisfaction of basic psychological needs

Self-determination was evaluated using a shorter Spanish adaptation [46] of the Basic Needs Satisfaction in General Scale [47]. This instrument consists of 16 items grouped into three subscales: a three-item subscale that evaluates Autonomy (e.g., ‘here I feel that I am free to decide for myself how to do my work’); a six-item subscale that assesses Competence (e.g., ‘People here tell me that I am good at what I do’); and a seven-item subscale that evaluates Relatedness (e.g., ‘The people around me here care about me’). Cronbach’s alpha reached values of .63, .62, and .78, respectively. Although these first two values were below .70, they were considered valid for analyses according to Hair et al.’s criterion [48].

### Data analysis

Scale scores were first standardized by converting them to z scores, thus ensuring comparable metrics of all variables. Descriptive analyses and z-order correlations of the analyzed factors were then computed.

Second, we classified the participants according to their 33rd and 66th percentile scores in each outcome (compassion satisfaction, compassion fatigue, and post-traumatic growth). This allowed better identifying the most extreme groups within the sample, as well as the protective factors that differentiate between those who showed a better psychological response in each outcome (i.e., lower compassion fatigue and higher compassion satisfaction and post-traumatic growth) and those who did not. To be precise, volunteers were classified as being ‘low’ (those who scored below the 33rd percentile), ‘medium’ (between the 33rd and the 66th percentile), or ‘high’ (higher than the 66th percentile) for each outcome. In this regard, we assumed that participants could be in different groups for each outcome. Moreover, we did not rule out possible nonlinear relationships between variables, since some evidence suggests curvilinear or non-linear links between PTG and psychological adjustment [21].

Third, after testing the homogeneity of the variances using Levene’s test, univariate analyses of variance (ANOVAs) with post-hoc Bonferroni tests were separately conducted for each outcome to evaluate potential differences between the groups. Welch’s test and the Games-Howell test were used in the cases in which variances were not homogeneous.

Finally, binary logistic regressions were conducted separately to identify the best predictors of the most extreme (low and high) levels of each outcome. Age and time spent volunteering were also included in the analyses as potential confounders.

## Results

### Descriptive analyses

Table 1 shows the descriptive statistics and z-order correlations between all the variables examined. The two self-compassionate strategies ultimately analyzed (self-kindness and mindfulness) were positively correlated with compassion satisfaction and post-traumatic growth and negatively correlated with compassion fatigue. Isolation and over-identification were positively correlated with compassion fatigue and negatively correlated with compassion satisfaction. In addition, isolation was negatively correlated with post-traumatic growth. Finally, satisfaction of the three basic psychological needs was positively correlated with compassion satisfaction and post-traumatic growth, whereas satisfaction of autonomy and relatedness, but not competence, was negatively correlated with compassion fatigue.

**Table 1:** Z-order correlations and descriptive statistics for the analyzed factors.

	1	2	3	4	5	6	7	8	9	M	SD
1. Compassion Fatigue										2.01	0.52
2. Compassion Satisfaction	.010									4.60	0.45
3. Post-traumatic Growth	.044	.389**								3.13	0.79
4. Self-kindness	-.155*	.316**	.266**							4.11	0.94
5. Mindfulness	-.185**	.190**	.156*	.516**						4.20	0.86
6. Isolation	.226**	-.175*	-.143*	-.373**	-.466**					2.12	1.09
7. Over-identification	.319**	-.189**	-.072	-.408**	-.468**	.697**				2.12	1.13
8. Autonomy	-.149*	.369**	.182**	.221**	.291**	-.221**	-.176*			5.56	1.16
9. Competence	-.112	.507**	.195**	.179**	.230**	-.291**	-.228**	.521**		5.43	1.06
10. Relatedness	-.164*	.457**	.254**	.218**	.262**	-.271**	-.239**	.625**	.760**	5.68	0.91

* p < .05.

** p < .01.

Moreover, age was negatively related to post-traumatic growth (*r* = -.17, *p* ≤ .05) but was not associated with compassion satisfaction or compassion fatigue. Age was also related to less isolation (*r* = -.21, *p* ≤ .01) and over-identification (*r* = -.27, *p* ≤ .01) and more mindfulness awareness (*r* = .19, *p* ≤ .01). The time spent volunteering was not significantly related to any of the variables analyzed.

### Volunteers with different levels of compassion fatigue

ANOVAs and post-hoc comparisons for each outcome are shown in the following three tables. In Table 2, the unhealthy strategies (isolation and over-identification) and mindfulness awareness stand out as the factors that allowed discriminating between the levels of compassion fatigue. In comparison to the other groups, the volunteers who indicated the highest level of compassion fatigue were also the ones who reported more frequent use of non-self-compassionate strategies (isolation and over-identification), which partially supported Hypothesis 2. By contrast, those who scored lowest in compassion fatigue reported higher mindfulness awareness than the highest group, partially supporting Hypothesis 1. The effect sizes were small, except for the differences found in over-identification, which reached a medium effect size.

**Table 2:** ANOVA comparing the participants’ scores on self-compassion and self-determination according to their level of compassion fatigue.

Predictive variables	Low (L)		Medium (M)		High (H)				Post-hoc		
	*M*	*SD*	*M*	*SD*	*M*	*SD*	*F*(2,208)	ղ^2^	*L-M*	*L-H*	*M-H*
Self-kindness	0.09	1.07	0.11	0.93	-0.24	0.97	2.77	0.03	-	-	-
Mindfulness	0.25	0.83	-0.08	1.18	-0.18	0.89	3.61*	0.03	0.33	0.43*	0.10
Isolation	-0.31	0.88	0.12	1.09	0.21	0.95	5.78**	0.05	-0.43*	-0.52**	-0.09
Over-identification	-0.40	0.77	0.08	1.08	0.35	0.97	13.32^w^***	0.10	-0.49**	-0.75***	-0.27
Autonomy	0.17	1.03	0.01	0.92	-0.20	1.04	2.38	0.02	-	-	-
Competence	0.02	1.14	0.13	0.85	-0.17	0.98	1.63	0.01	-	-	-
Relatedness	0.10	1.06	0.11	0.89	-0.23	1.02	2.59	0.02	-	-	-

*Note*.

^W^ Welch’s test

*p ≤ .05.

** *p* ≤ .01.

*** *p* ≤ .001. Means and standard deviations are z scores.

### Volunteers with different levels of compassion satisfaction

As shown in Table 3, the volunteers high in compassion satisfaction reported a higher satisfaction of the three basic psychological needs (autonomy, competence, and relatedness) than the other groups. In addition, they showed a higher use of healthy strategies (self-kindness and mindfulness) and a lower use of unhealthy ones (isolation and over-identification) than those lowest in compassion satisfaction. These results support the three hypotheses, with effect sizes larger than in the other two outcomes.

**Table 3:** ANOVA comparing the participants’ scores on self-compassion and self-determination according to their level of compassion satisfaction.

Predictive variables	Low (L)		Medium (M)		High (H)				Post-hoc		
	*M*	*SD*	*M*	*SD*	*M*	*SD*	*F*(2,208)	ղ^2^	*L-M*	*L-H*	*M-H*
Self-kindness	-0.58	1.05	0.16	0.84	0.29	0.92	14.40***	0.14	-0.75***	-0.87**	-0.12
Mindfulness	-0.29	0.98	-0.06	0.98	0.28	0.96	5.92**	0.06	-0.23	-0.58**	-0.35
Isolation	0.33	1.03	-0.06	0.88	-0.20	1.03	4.97**	0.05	0.39	0.53**	0.14
Over-identification	0.32	1.02	-0.04	0.90	-0.20	1.03	4.86**	0.05	0.39	0.53**	0.14
Autonomy	-0.44	1.04	-0.03	0.92	0.37	0.90	11.44***	0.11	-0.41*	-0.82***	-0.41*
Competence	-0.58	1.05	-0.06	0.82	0.50	0.85	20.21***	0.19	-0.52**	-1.08**	-0.57***
Relatedness	-0.57	1.03	0.06	0.90	0.38	0.87	15.71***	0.15	-0.64***	-0.95***	-0.32

*Note*.

^W^ Welch’s test

*p ≤ .05.

** *p* ≤ .01.

*** *p* ≤ .001. Means and standard deviations are z scores.

### Volunteers with different levels of post-traumatic growth

The volunteers highest in post-traumatic growth showed a higher satisfaction with the basic psychological needs and a higher self-kindness than the other groups, but no other significant differences (Table 4). These results contribute to supporting Hypothesis 3 and, partially, Hypothesis 1. In both cases, the effect sizes ranged from small to medium.

**Table 4:** ANOVA comparing the participants’ scores on self-compassion and self-determination according to their level of post-traumatic growth.

Predictive variables	Low (L)		Medium (M)		High (H)				Post-hoc		
	*M*	*SD*	*M*	*SD*	*M*	*SD*	*F*(2,208)	ղ^2^	*L-M*	*L-H*	*M-H*
Self-kindness	-0.29	1.13	-0.16	0.94	0.48	0.74	15.47^W^***	0.11	-0.13	-0.77***	-0.64***
Mindfulness	-0.12	1.11	-0.12	1.00	0.26	0.83	3.46	0.03	-	-	-
Isolation	0.16	1.09	0.05	0.99	-0.21	0.88	2.45	0.02	-	-	-
Over-identification	0.11	1.08	0.01	0.93	-0.11	0.99	0.82	0.01	-	-	-
Autonomy	-0.24	1.04	-0.07	1.05	0.32	0.80	5.85**	0.05	-0.17	-0.56**	-0.40*
Competence	-0.18	1.09	-0.10	0.92	0.30	0.92	4.81**	0.04	-0.08	-0.49*	-0.41*
Relatedness	-0.33	0.95	-0.03	0.99	0.35	0.94	8.35***	0.07	-0.30	-.69***	-0.39

*Note*.

^W^ Welch’s test

*p ≤ .05.

** *p* ≤ .01.

*** *p* ≤ .001. Means and standard deviations are z scores.

### Predictors of the most extreme groups

Table 5 shows the results of the binary logistic regressions computed for each of the outcomes. Low compassion fatigue was significantly predicted by less use of over-identification as shown by the adequate specificity of the first model. In the second model, compassion satisfaction was predicted by self-kindness and the satisfaction of feeling competent. Finally, the third model revealed that greater self-kindness and satisfaction of the need for relationships, as well as younger age, predicted higher post-traumatic growth.

**Table 5:** Binary logistic regression to predict the highest and lowest levels of the analyzed outcomes.

Predictors	Compassion Fatigue						Compassion Satisfaction						Post-traumatic Growth (PTG)					
	B	*SE*	*Wald’s test*	*p*	Odds Ratio	95% CI	B	*SE*	*Wald’s test*	*p*	Odds Ratio	95% CI	B	*SE*	*Wald’s test*	*p*	Odds Ratio	95% CI
Self-kindness							.819	.218	14.08	< .001	2.27	[1.48, 3.48]	.903	.242	13.91	< .01	2.47	[1.53, 3.97]
Over-identification	.972	.226	18.46	< .001	2.64	[1.70, 4.12]												
Competence							1.15	.243	22.50	< .001	3.16	[1.96, 5.09]						
Relatedness													.588	.221	7.11	< .01	1.80	[1.17, 2.77]
Age													-.050	.019	6.86	< .01	0.95	[.917,.988]
	χ^2^ (1) = 23.02, *p* < .001						χ^2^ (2) = 55.71, *p* < .001						χ^2^ (3) = 37.31, *p* < .001					
	68.0% were correctly classified (79.2% of the true-negatives and 55.0% of the true-positives)						79.0% were correctly classified (73.3% of the true-negatives and 83.3% of the true-positives)						70.2% were correctly classified (66.2% of the true-negatives and 74.2% of the true-positives)					

B = Regression coefficients. SE = standard error. CI = confidence interval.

## Discussion

The aim of this cross-sectional study was twofold. The first objective was to examine to what extent self-compassion (as shown by a higher frequency of self-compassionate strategies and a lower frequency of non-self-compassionate ones) and self-determination (sustained by the satisfaction, through volunteering, of basic psychological needs) would contribute to differentiating between volunteers with distinct levels of compassion fatigue, compassion satisfaction, and post-traumatic growth. The findings revealed that self-compassion and self-determination contribute differently to discriminating between the volunteers who are low, medium, and high in each outcome. While compassion satisfaction aligned entirely with the hypotheses, compassion fatigue and post-traumatic growth only did so partially.

As expected, the volunteers who indicated being more affected by compassion fatigue reported a more frequent use of non-self-compassionate strategies and a lower mindfulness awareness than those less affected. However, they did not show differences in self-kindness or satisfaction of basic psychological needs. The two ways of negatively relating to oneself (i.e., isolation and over-identification) were also found in those with lower compassion satisfaction. Therefore, prevention of compassion fatigue requires one to focus on retraining these unhealthy strategies and broadening volunteers’ perspective in the face of difficult situations.

Moreover, the self-compassionate and non-self-compassionate strategies were negatively correlated to each other, thus supporting the idea that these strategies reflect opposing manners of considering one’s own difficulties or failures [30, 37]. For instance, people who focus on their personal suffering usually have more difficulties viewing their experience from a broader perspective. In this study, those most affected by compassion fatigue also indicated a lower mindfulness awareness, which hinders one from keeping adverse experiences in perspective. Overall, these results also are consistent with previous research indicating that negative affect and narrowed thinking impair ability for self-compassion, thus increasing the risk of psychopathology [34, 49, 50].

The volunteers with distinct levels of compassion satisfaction did show the expected differences in all factors analyzed, i.e., higher use of self-compassionate strategies and lower use of non-self-compassionate ones, as well as greater satisfaction of the basic psychological needs. Consistently, self-compassion has proven to increase life satisfaction and positive mental health outcomes [35, 37]. From a different theoretical approach, self-determination has also been linked to more effective performance, psychological health, and growth [39, 40]. The findings of this study suggest that intervention to promote self-determination could increase compassion satisfaction but would not reduce compassion fatigue. By contrast, intervention to prevent unhealthy strategies could be effective for both objectives.

Moreover, compassion fatigue and compassion satisfaction were not correlated with each other. This means that both responses to people’s suffering may be experienced simultaneously [4], but it does not support that increasing compassion satisfaction would help balance the ratio between the two dimensions [5]. Although the mechanisms underlying compassion fatigue are still under discussion, this result is compatible with the idea that compassion fatigue is not due to excessive compassion, but to a strong aversive response in the face of others’ suffering [29]. In this regard, it seems necessary to guide volunteers to differentiate between an unhealthy concern and compassion toward others [28].

With the exception of self-kindness, the results did not lend support to the association between self-compassion and post-traumatic growth. However, post-traumatic growth did require volunteers to feel autonomous, competent, and related to other significant people doing their work. This latter result is consistent with a proactive way to face difficulties. In Yehuda’s words, resilience involves ‘a conscious effort to move forward in an insightful integrated positive manner’ (p. 3) [51]. Beyond being a positive adaptation that leads to pre-trauma levels of functioning [19], resilience may involve thriving for some people [20]. In addition to an effortful attitude, our findings reveal that post-traumatic growth (an indicator of thriving) is more likely when the volunteers’ basic psychological needs are satisfied. This also suggests that volunteers will be better prepared to navigate distressing experiences. Drawing on the self-determination approach, this means possessing an autonomous self and not depending on unstable achievements or conditional approval. Consistently, previous research has indicated that volunteers’ resilience may benefit from a sense of self-efficacy [52], psychological endurance, and organization support [16, 17]. All these characteristics may be targeted for intervention.

The second objective was to identify the predictors that allow volunteers to be classified into the most extreme groups (i.e., those who scored lowest and highest in each outcome). The first model showed an adequate level of specificity (true-negative rate), which points to the finding that less frequent use of over-identification is associated with a lower level of compassion fatigue. Among the self-compassionate strategies, self-kindness allowed classifying those who scored highest and lowest in the other two outcomes. As for satisfaction of basic psychological needs, while competence predicted compassion satisfaction, relatedness predicted post-traumatic growth. According to these results, increasing self-kindness and decreasing over-identification are eligible for the design of self-care training. Moreover, nonprofit organizations can improve volunteers’ well-being by fostering the satisfaction of competence and relatedness. Finally, age deserves a separate mention. It is negatively associated with post-traumatic growth, but not with compassion satisfaction or compassion fatigue. This could indicate that older volunteers have developed a previous adaptation throughout their lives or in the face of the difficulties of volunteering. This is suggested by negative correlations between age and non-compassionate strategies, as well as positive correlations between age and mindfulness awareness.

### Limitations and future directions of research

This cross-sectional study was conducted shortly after the end of the lockdown in Spain (March-June 2020), offering a picture of a relatively good moment during this pandemic, when the first wave of the outbreak had been defeated. In that moment, people felt relief, which likely facilitated the obtaining of good psychological measures among volunteers (higher levels of compassion satisfaction and thriving and lower compassion fatigue). Therefore, knowing how self-compassion and self-determination could affect each of the outcomes over time would require a longitudinal design.

Studies carried out in Spain have confirmed that mental health, both in the population and in health professionals, is being negatively affected by the pandemic [53, 54]. In addition, since we expected volunteers to be exhausted after lockdown, we wanted to design a single short survey. For those reasons, we only measured compassion fatigue as an indicator of psychological distress. However, it would be necessary to test changes in distress over time to demonstrate that the factors analyzed really are protective in the face of continued adversity.

The small effect size of most of the significant differences found limits the generalizability of the results. Hence, it is necessary to be cautious in interpreting the results until the study can be replicated with a much larger sample. However, despite the rules of thumb for interpreting effect sizes of ANOVAs [55], the findings may have clinical significance, since they may help improve volunteers’ quality of life and labor [56]. In a similar vein, we did not collect information on other variables that may have affected the results (e.g., the number of people that volunteers helped per day or the experience level). Therefore, this should be controlled in future studies.

While the population was under the lockdown, volunteers were actively contributing to helping suffering people. This situation likely allowed volunteers to feel useful and agentic. However, Spain was to suffer new waves of the outbreak much earlier than expected. Therefore, we do not know if volunteers who reported thriving were able to maintain their strength in the face of new difficulties.

Finally, another potential limitation of this study could come from a social desirability bias, which could explain the low reliability shown by some measures, such as the compassion fatigue scale. Although we highlighted the independence of the research team, the invitations to respond to the survey were sent by the staff of the organization. Therefore, we cannot rule out that volunteers’ responses were affected by a positive response bias.

### Implications for intervention

Previous studies conducted so far have drawn attention to the fact that the psychosocial stressors created by the pandemic may exacerbate existing psychopathology and cause negative direct psychological consequences [57–60]. In this context, volunteers are likely to have experienced the same threats as other groups (fear of contagion, losing their job, or loss of loved ones). In addition, they were dealing with the suffering of the most vulnerable people. Considering these premises, the current study points to specific factors that may be targeted for improving their well-being.

According to the findings, the most urgent task is to offer guidance to volunteers to increase their self-compassion and reduce unhealthy ways of relating to themselves. This would contribute not only to reducing the risk of compassion fatigue, but also to alleviating the psychological burden they may experience as citizens. Assuming that self-compassion can be acquired through practice [11, 36, 61], nonprofit organizations need to target this protective factor in their training programs. The precise mechanisms that explain the protective effects of self-compassion are unclear, but some potential candidates could contribute to improving it. Thus, emotional regulation and emotional awareness [62, 63] or changing beliefs in failure [64] are thought to be helpful for this aim.

Bolstering resilience is among the most promising interventions for reducing compassion fatigue in professionals [65]. Although specific studies on volunteers’ resilience are still scarce, research on resilience in different populations points to specific strengths that predict well-functioning and thriving [66, 67]. In addition, our findings suggest that some of these strengths could be relevant because they contribute to the satisfaction of basic psychological needs. For instance, strengths such as self-efficacy, psychological endurance, or purpose have been linked to volunteers’ resilience [10, 11] and they could facilitate the satisfaction of autonomy and competence. Moreover, organizational support seems clearly linked to the need of relatedness, although it could also be necessary for the satisfaction of competence. In fact, training was the request made most often to the Red Cross. Therefore, the association between these strengths and the satisfaction of basic psychological needs requires further research, since it could help design training to enhance resilience.

According to some studies, resilient people have adapted better to lockdown [54]. Resilience, however, is more than the absence of psychological symptoms, and we need to identify modifiable factors that may be targeted by intervention. Feeling capable of helping others in times of crisis may be a source of compassion satisfaction and thriving [4, 5]. However, this requires a supporting organizational context that allows volunteers to feel autonomy and connection to others within the organization as well as consider themselves prepared for helping. As volunteers indicated, they need be trained to face the crisis and have enough resources to manage the situation.

## Conclusion

With some exceptions [59, 66], research has focused on the negative mental health consequences of the SARS-CoV-2 pandemic. By contrast, this study contributes to the knowledge of volunteers’ self-care and thriving, offering guidance to nonprofit organizations for preventing the negative consequences of exposure to highly stressful events during the worst moments of the pandemic. The findings reveal the importance of reducing non-self-compassionate strategies, especially over-identification, to prevent compassion fatigue. In addition, they indicate the need to increase self-kindness as a healthy strategy, and create the conditions that allow the needs of autonomy, competence, and relatedness to be satisfied. In this way, volunteers would benefit from higher compassion satisfaction and thriving.

## Supporting information

S1 File (XLSX)
